# Prevalence and health outcomes of combustible cigarettes and noncombustible nicotine or tobacco products: a nationwide cross-sectional study in South Korea

**DOI:** 10.1016/j.pmedr.2025.103202

**Published:** 2025-08-10

**Authors:** Tae Hyeon Kim, Yeona Jo, Jaewon Kim, Krishna Prasad Acharya, Hanseul Cho, Ho Geol Woo, Jiyoung Hwang, Dong Keon Yon

**Affiliations:** a Center for Digital Health, Medical Science Research Institute, Kyung Hee University Medical Center, Kyung Hee University College of Medicine, Seoul, South Korea; b Department of Medicine, Kyung Hee University College of Medicine, Seoul, South Korea; c Department of Precision Medicine, Kyung Hee University College of Medicine, Seoul, South Korea; d Animal Disease Investigation and Control Division, Department of Livestock Services, Hariharbhawan, Lalitpur, Nepal; e Department of Epidemiology, Harvard T.H. Chan School of Public Health, Boston, MA, USA; f Department of Neurology, Kyung Hee University Medical Center, Kyung Hee University College of Medicine, Seoul, South Korea; g Department of Pediatrics, Kyung Hee University Medical Center, Kyung Hee University College of Medicine, Seoul, South Korea

**Keywords:** Combustible cigarette, Health outcomes, Non-combustible nicotine or tobacco product, South Korea, Trend

## Abstract

**Objective:**

Limited evidence exists on type of tobacco use changes and differential health outcomes following the introduction of non-combustible nicotine or tobacco products (NNTPs). Therefore, this study aimed to evaluate the characteristics, trends, and health outcomes of exclusive combustible cigarette users and dual users of combustible cigarette and NNTP.

**Methods:**

This study analyzed data from the Korea National Health and Nutrition Examination Survey from 2013 to 2021 (*n* = 18,763). Individuals aged ≥19 years were included in the study. Type of tobacco use were categorized into exclusive combustible cigarette users or dual users. Health outcomes assessed included stroke, angina pectoris, hypertension, dyslipidemia, type 2 diabetes, rheumatoid arthritis, allergic rhinitis, atopic dermatitis, and depression. Prevalence trends were estimated using weighted complex survey analysis, with results presented as weighted prevalence and 95 % confidence intervals (CIs). Health outcomes by type of tobacco use were evaluated using multivariable logistic regression, providing adjusted odds ratios (aORs) with 95 % CIs.

**Results:**

Exclusive combustible cigarette user showed a decreasing trend, while dual exhibited a showed increase. This shift from exclusive combustible cigarette user to dual user was particularly evident among younger, highly educated, and higher-income individuals. Compared to exclusive combustible cigarette users, dual users exhibited significantly associations with depression (aOR, 1.50 [95 % CI, 1.19–1.89]), atopic dermatitis (1.39 [1.10–1.74]), and allergic rhinitis (1.30 [1.14–1.49]).

**Conclusions:**

The prevalence of dual use is increasing and is associated with a higher risk of depression, atopic dermatitis, and allergic rhinitis.

## Highlights

• A shift from exclusive cigarette use to dual use is increasingly observed in Korea.
• Young, educated, and high-income individuals are more likely to be dual users.
• Dual users show higher risks of depression, allergic rhinitis, and atopic dermatitis.

## 1 Introduction

Various national initiatives have been implemented to reduce the health risks and societal burden associated with smoking (Chido-Amajuoyi et al., 2020). These efforts include increasing taxes on tobacco products, expanding smoke-free zones, and providing smoking cessation education and support through public health centers (Jegasothy and Markham, 2024). Consequently, public perception of conventional combustible cigarettes has deteriorated, with many individuals feeling social and financial pressure to quit. In this context, noncombustible nicotine or tobacco products (NNTP) have emerged as an alternative, perceived by some as a safer option than combustible cigarettes (Jackson et al., 2024). Many tobacco users consider NNTP as a transitional tool, planning to quit smoking by first switching to NNTP before eventually stopping all nicotine use (Huang et al., 2019).

The prevalence of dual use, referring to individuals who use both combustible cigarette and NNTP, has reportedly increased significantly (Martinez et al., 2021). However, NNTP have been associated with unique pulmonary conditions, such as e-cigarette or vaping product use-associated lung injury, raising concerns about their safety (Kalininskiy et al., 2019). Furthermore, current evidence suggests that NNTPs are associated with acute vasoconstriction, impaired exercise tolerance, and other cardiovascular symptoms shortly after exposure, indicating a potential influence on various health outcomes (Jin et al., 2021). Due to the relatively recent introduction of these products, the long-term health effects of NNTP and the potential synergistic effects of dual use remain uncertain (Drope et al., 2017).

Therefore, this study aimed to evaluate the characteristics, trends, and health outcomes of exclusive combustible cigarette users and dual users of combustible cigarette and NNTP, using a nationwide cross-sectional survey in South Korea from 2013 to 2021. Differences in health outcomes across types of tobacco use were analyzed to provide further context to the ongoing discussion regarding the relative harms of dual use compared to exclusive combustible cigarette use. Given the increasing use of NNTPs in South Korea since their introduction (e-cigarettes in 2007, heated tobacco products in 2017) (Kim and Cho, 2020), analyzing trends in dual use and associated health risks using Korean data provides valuable evidence to inform public health policies and support individuals in making informed decisions regarding smoking cessation and NNTP use.

## 2 Methods

### 2.1 Data source

This study utilized data from the Korea National Health and Nutrition Examination Survey (KNHANES), conducted by the Korea Disease Control and Prevention Agency (KDCA). As KNHANES provides only sampling weights, analyses were conducted using complex survey procedures incorporating sampling weights, stratification, and clustering (Park et al., 2023). To compare prevalence and trends of exclusive combustible cigarette users and dual users of combustible cigarette and NNTP, we analyzed data from the KNHANES conducted between 2013 and 2021 (*N* = 44,879), during which NNTP-related survey questions were included. Individuals under 19 years of age (*n* = 1255) and those with missing data on key covariates (*n* = 338) were excluded using a complete-case analysis. Furthermore, non-tobacco users (*n* = 24,491) and exclusive NNTP users (*n* = 32) were excluded to restrict the analysis to exclusive combustible cigarette users and dual users. The final analytic sample comprised 16,621 exclusive combustible cigarette users and 2142 dual users in this study (**Fig. S1**).

The KNHANES database was anonymized, and all study participants provided informed consent for participation. The study protocol was approved by the Institutional Review Board of the KDCA (approval numbers: 2007-02CON-04-P, 2008-04EXP-01-C, 2009-01CON-03-2C, 2010-02CON-21-C, 2011-02CON-06-C, 2012-01EXP-01-2C, 2013-07CON-03-4C, 2013-12EXP-03-5C, 2018-01-03-P-A, 2018-01-03-C-A, 2018-01-03-2C-A, and 2018-01-03-5C-A) and by ethical review from the Institutional Review Board of Kyung Hee University. In addition, this study was conducted in compliance with the principles of the Declaration of Helsinki.

### 2.2 Exposure

The primary exposure variable was types of tobacco use, categorized into two groups: exclusive combustible cigarette users and dual users of combustible cigarette and NNTP. Participants were classified as combustible cigarette users if they did not respond “never used” to the question, “How many combustible cigarettes have you use in your lifetime?”, and answered “every day” or “sometimes” to the question, “Do you currently use combustible cigarettes?”. Users of NNTPs were defined as those who answered “every day” or “sometimes” to the question, “Do you currently use heated tobacco products?”, or “yes” to “Have you used nicotine-containing liquid e-cigarettes in the past month?”. Participants meeting both criteria were categorized as dual users, while those meeting neither were classified as non-tobacco users. Prevalence trends for exclusive combustible cigarette users and dual users were assessed using data from all participants. However, for the comparison of characteristics between exclusive combustible cigarette users and dual users, and the examination of their associations with health outcomes, the analysis excluded exclusive NNTP users (*n* = 32) and non-tobacco users (*n* = 24,491), to ensure a focused and distinct evaluation of their characteristics.

### 2.3 Health outcomes

The primary outcomes were cardiovascular diseases (stroke and angina pectoris), metabolic disorders (hypertension, dyslipidemia, and type 2 diabetes), inflammatory diseases (rheumatoid arthritis, allergic rhinitis, and atopic dermatitis), and mental health conditions (depression) (Reitsma et al., 2021). Health outcomes were chosen from variables that were consistently assessed in the survey during the study period. Disease status was assessed using participants' responses to the question, “Have you ever been diagnosed with the disease by a doctor?”

### 2.4 Covariates

Demographic covariates included age (19–39, 40–64, and ≥ 65 years), sex (male and female), residence (urban and rural) (Park et al., 2023), cigarette consumption amount (<10, 10–19 and ≥ 20 cigarettes per day), education level (elementary school or lower education, middle school, high school, and college or higher education), household income (lowest quartile, second quartile, third quartile, and highest quartile), body mass index (BMI) group. In this study, BMI categories followed the Asian-Pacific guidelines: underweight (<18.5 kg/m^2^), normal weight (18.5–22.9 kg/m^2^), overweight (23.0–24.9 kg/m^2^), and obese (≥25.0 kg/m^2^) (Park et al., 2023).

### 2.5 Statistical analysis

Prevalence and trend were estimated by employing weighted complex survey procedures, accounting for stratification, clustering, and sampling weights to generate estimates with 95 % confidence intervals (CIs). Chi-square tests were conducted to assess whether the distribution of specific variables differed significantly across categories within each baseline characteristic variable. To examine differences in baseline characteristics between exclusive combustible cigarette users and dual users, we applied a multivariable logistic regression model in which type of tobacco use was the dependent variable, and all baseline characteristics were entered as independent variables; weighted odds ratios (wORs) with 95 % CIs were calculated. The association between types of tobacco use and health outcomes was assessed using adjusted odds ratios (aORs) with 95 % CIs, derived from multivariable logistic regression, adjusting for age, sex, smoking amount, education level, household income, and BMI group. Statistical significance was defined as *p* < 0.05 using two-sided tests (Hong et al., 2024). Statistical analyses were conducted using SAS software (version 9.4, SAS Institute, Cary, NC, USA).

## 3 Results

### 3.1 Trend in types of tobacco use

**Fig. 1:**
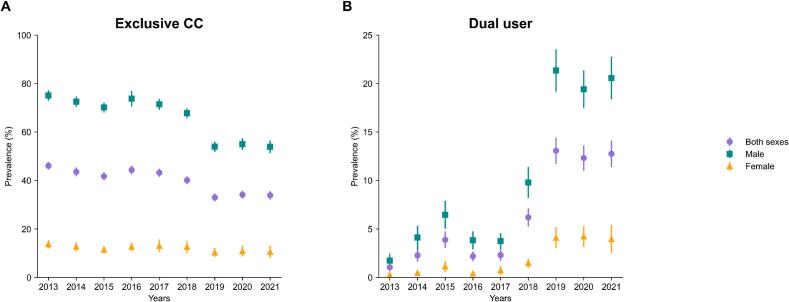
National trends in the weighted prevalence of (A) exclusive combustible cigarette users and (B) dual users among Korean adults in the Korea National Health and Nutrition Examination Survey, 2013–2021. Error bars represent 95 % confidence intervals. Abbreviations: CC, combustible cigarette.

As of 2021, exclusive combustible cigarette users comprised 33.90 % (95 % CI, 32.18–35.62) and dual users comprised 12.76 % (95 % CI, 11.39–14.13). The prevalence of exclusive combustible cigarette users exhibited a decreasing trend from 46.11 % (95 % CI, 44.53–47.69) in 2013, whereas the prevalence of dual users showed a concomitant increasing trend from 1.05 % (95 % CI, 0.64–1.46) in 2013 (**Tables S1 and S2**). Fig. 1 represents these prevalence trends, indicating that the rise in dual user prevalence coincided with the decline in exclusive combustible cigarette user prevalence. Notably, the prevalence of dual users consistently remained above 10 % from 2019 onwards. Furthermore, a marked disparity in smoking prevalence by sex is evident.

### 3.2 Characteristics of exclusive combustible cigarette users and dual users

To understand the apparent shift in prevalence from exclusive combustible cigarette users to dual users, a comparative analysis was performed between these two groups, excluding exclusive NNTP users and non-tobacco users (Table 1). Among exclusive combustible cigarette users, the 40–64 age group was most prevalent (53.11 % [95 % CI, 52.16–54.06]), whereas dual users were more frequently aged 19–39 (62.52 % [95 % CI, 60.08–64.97]). Both exclusive combustible cigarette users (85.57 % [95 % CI, 84.95–86.18]) and dual users (85.84 % [95 % CI, 84.33–87.35]) were predominantly male. A higher proportion of exclusive combustible cigarette users resided in urban areas (83.39 % [95 % CI, 81.63–85.15]), a similar pattern also observed among dual users (89.66 % [95 % CI, 87.49–91.84]).

**Table 1:** Baseline characteristics of exclusive combustible cigarette users and dual users in South Korea from Korea National Health and Nutrition Examination Survey, 2013–2021.

Characteristic	Exclusive combustible cigarette users (*n* = 16,621)		Dual users (*n* = 2142)	
	Unweighted, n (%)	Weighted, % (95 % CI)	Unweighted, n (%)	Weighted, % (95 % CI)
Age group, years				
19–39	3967 (23.87)	31.50 (30.47 to 32.53)	1251 (58.40)	62.52 (60.08 to 64.97)
40–64	8340 (50.18)	53.11 (52.16 to 54.06)	817 (38.14)	35.84 (33.42 to 38.26)
≥65	4314 (25.96)	15.39 (14.77 to 16.02)	74 (3.45)	1.64 (1.22 to 2.05)
Sex				
Male	14,001 (84.24)	85.57 (84.95 to 86.18)	1789 (83.52)	85.84 (84.33 to 87.35)
Female	2620 (15.76)	14.43 (13.82 to 15.05)	353 (16.48)	14.16 (12.65 to 15.67)
Region of residence				
Urban	13,312 (80.09)	83.39 (81.63 to 85.15)	1877 (87.63)	89.66 (87.49 to 91.84)
Rural	3309 (19.91)	16.61 (14.85 to 18.37)	265 (12.37)	10.34 (8.16 to 12.51)
Smoking amount, cigarettes/day				
<10	4547 (27.36)	27.99 (27.17 to 28.80)	518 (24.18)	24.93 (22.90 to 26.96)
11–19	5897 (35.48)	36.71 (35.82 to 37.60)	998 (46.59)	46.36 (44.01 to 48.71)
≥20	6177 (37.16)	35.31 (34.45 to 36.17)	626 (29.23)	28.71 (26.63 to 30.79)
Educational level				
Elementary school or lower education	2161 (13.00)	8.68 (8.19 to 9.17)	31 (1.45)	0.93 (0.52 to 1.33)
Middle school	1909 (11.49)	9.15 (8.63 to 9.67)	73 (3.41)	2.69 (1.95 to 3.43)
High school	5356 (32.22)	32.77 (31.83 to 33.71)	591 (27.59)	27.30 (25.02 to 29.59)
College or higher education	7195 (43.29)	49.40 (48.23 to 50.58)	1447 (67.55)	69.09 (66.73 to 71.45)
Household income				
Lowest quartile	3048 (18.34)	14.38 (13.65 to 15.10)	181 (8.45)	7.57 (6.31 to 8.83)
Second quartile	4251 (25.58)	24.53 (23.62 to 25.43)	470 (21.94)	20.73 (18.66 to 22.80)
Third quartile	4646 (27.95)	30.30 (29.31 to 31.28)	686 (32.03)	33.72 (31.19 to 36.26)
Highest quartile	4676 (28.13)	30.80 (29.66 to 31.95)	805 (37.58)	37.98 (35.27 to 40.69)
BMI group^⁎^				
Underweight	539 (3.24)	3.22 (2.90 to 3.54)	66 (3.08)	2.94 (2.14 to 3.73)
Normal weight	5501 (33.10)	32.70 (31.85 to 33.54)	624 (29.13)	28.67 (26.54 to 30.80)
Overweight	4173 (25.11)	24.65 (23.87 to 25.43)	476 (22.22)	23.03 (20.91 to 25.15)
Obese	6408 (38.55)	39.43 (38.55 to 40.31)	976 (45.56)	45.36 (42.99 to 47.73)
Marital status				
Married	14,065 (84.62)	79.04 (78.13 to 79.96)	1252 (58.45)	54.87 (52.14 to 57.60)
Unmarried	2556 (15.38)	20.96 (20.04 to 21.87)	890 (41.55)	45.13 (42.40 to 47.86)
Subjective health level				
High	4974 (29.93)	31.28 (30.43 to 32.12)	634 (29.60)	29.58 (27.42 to 31.73)
Middle	8542 (51.39)	51.75 (50.84 to 52.66)	1140 (53.22)	53.61 (51.23 to 55.99)
Low	3105 (18.68)	16.97 (16.29 to 17.66)	368 (17.18)	16.81 (15.03 to 18.59)
Subjective stress level				
High	4304 (25.90)	28.13 (27.30 to 28.97)	830 (38.75)	38.67 (36.20 to 41.14)
Middle	9508 (57.20)	57.33 (56.45 to 58.20)	1119 (52.24)	52.27 (49.83 to 54.70)
Low	2809 (16.90)	14.54 (13.92 to 15.16)	193 (9.01)	9.07 (7.70 to 10.43)
Alcohol consumption, days/month				
<1	4568 (27.48)	24.94 (24.18 to 25.70)	393 (18.35)	18.28 (16.41 to 20.15)
1–4	5661 (34.06)	36.07 (35.18 to 36.97)	880 (41.08)	41.11 (38.67 to 43.56)
≥5	6392 (38.46)	38.99 (38.11 to 39.87)	869 (40.57)	40.61 (38.26 to 42.96)

Abbreviations: BMI, body mass index; NNTP, noncombustible nicotine or tobacco product.

* According to Asian-Pacific guidelines, BMI is divided into four groups: underweight (<18.5 kg/m^2^), normal weight (18.5–22.9 kg/m^2^), overweight (23.0–24.9 kg/m^2^), and obese (≥25.0 kg/m^2^).

Table 2 presents the distribution of each characteristic variable between exclusive combustible cigarette users and dual users. Compared to exclusive combustible cigarette users, dual users were more likely to be younger, reside in urban areas, have higher educational attainment and income levels, be obese, unmarried, report poorer subjective health, experience higher stress, and consume alcohol more frequently. Dual users also exhibited significantly higher cigarette consumption. No significant difference was observed between the groups regarding sex.

**Table 2:** Association between sociodemographic and behavioral characteristics and type of tobacco use among Korean adults in the Korea National Health and Nutrition Examination Survey, 2013–2021.

Characteristic	Dual users vs. Exclusive combustible cigarette users
	Adjusted wOR (95 % CI)
Age group, years	
19–39	1.00
40–64	0.50 (0.43 to 0.58)
≥65	0.15 (0.11 to 0.20)
Sex	
Female	1.00
Male	1.10 (0.93 to 1.29)
Region of residence	
Urban	1.00
Rural	0.72 (0.58 to 0.90)
Smoking amount, cigarettes/day	
<10	1.00
11–19	1.69 (1.47 to 1.95)
≥20	1.50 (1.28 to 1.76)
Educational level	
Elementary school or lower education	1.00
Middle school	1.70 (1.00 to 2.88)
High school	2.85 (1.79 to 4.54)
College or higher education	3.44 (2.15 to 5.50)
Household income	
Lowest quartile	1.00
Second quartile	1.18 (0.94 to 1.49)
Third quartile	1.37 (1.09 to 1.70)
Highest quartile	1.46 (1.17 to 1.82)
BMI group^⁎^	
Underweight	1.00
Normal weight	1.17 (0.84 to 1.62)
Overweight	1.37 (0.97 to 1.92)
Obese	1.55 (1.11 to 2.16)
Marital status	
Married	1.00
Unmarried	1.74 (1.51 to 2.01)
Subjective health level	
High	1.00
Middle	1.18 (1.04 to 1.34)
Low	1.24 (1.03 to 1.48)
Subjective stress level	
High	1.00
Middle	1.18 (1.04 to 1.34)
Low	1.24 (1.03 to 1.48)
Alcohol consumption, days/month	
<1	1.00
1–4	1.13 (0.97 to 1.32)
≥5	1.24 (1.07 to 1.44)

Abbreviations: BMI, body mass index; CI, confidence interval; KNHANES, Korea National Health and Nutrition. Examination Survey; NNTP, noncombustible nicotine or tobacco product; wOR, weighted odds ratio.

* According to Asian-Pacific guidelines, BMI is divided into four groups: underweight (<18.5 kg/m^2^), normal weight (18.5–22.9 kg/m^2^), overweight (23.0–24.9 kg/m^2^), and obese (≥25.0 kg/m^2^).

† Multivariable logistic regression model is adjusted for age, sex, region of residence, smoking amount, educational level, household income, BMI, marital status, subjective health level, subjective stress level, and alcohol consumption.

### 3.3 Health outcomes associated with exclusive combustible cigarette users and dual users

**Fig. 2:**
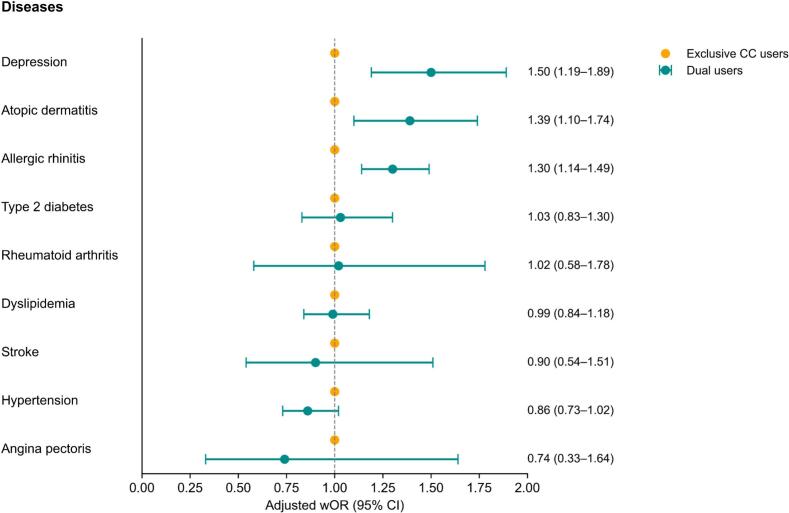
Association between tobacco use type and health conditions among Korean adults in the Korea National Health and Nutrition Examination Survey, 2013–2021. Abbreviations: BMI, body mass index; CC, combustible cigarette; CI, confidence interval; wOR, weighted odds ratio. † The model was adjusted for age, sex, smoking amount, educational level, household income, BMI, and smoking amount.

Fig. 2 presents the comparative results of various health outcomes between exclusive combustible cigarette users and dual users. Compared to exclusive combustible cigarette users, dual users showed significantly stronger associations with depression (aOR, 1.50 [95 % CI, 1.19–1.89]), atopic dermatitis (aOR, 1.39 [95 % CI, 1.10–1.74]), and allergic rhinitis (aOR, 1.30 [95 % CI, 1.14–1.49]). Conversely, no significant differences in association were observed between two groups for type 2 diabetes (aOR, 1.03 [95 % CI, 0.83–1.30]), rheumatoid arthritis (aOR, 1.02 [95 % CI, 0.58–1.78]), dyslipidemia (aOR, 0.99 [95 % CI, 0.84–1.18]), stroke (aOR, 0.90 [95 % CI, 0.54–1.51]), hypertension (aOR, 0.86 [95 % CI, 0.73–1.02]), and angina pectoris (aOR, 0.74 [95 % CI, 0.33–1.64]) (**Table S3**).

## 4 Discussion

This study investigated the prevalence trends of exclusive combustible cigarette users and dual users, and compared the characteristics and associations with various health outcomes for each group. Dual user prevalence, initially low, gradually increased and has remained above 10 % since 2019. The increase in dual user prevalence coincided with a decrease in exclusive combustible cigarette user prevalence, suggesting a shift from exclusive combustible cigarette use to dual use. Compared to exclusive combustible cigarette users, dual users were characterized by being younger, residing in urban areas, having higher education levels, and income quintiles, being more frequently obese and unmarried, reporting lower subjective health status and higher stress levels, and consuming more alcohol. Dual users showed significantly stronger associations with depression, atopic dermatitis, and allergic rhinitis compared to exclusive combustible cigarette users.

The observed decline in exclusive combustible cigarette users and the concurrent rise in dual users represents a global pattern. In the US, a significant association has been identified between the decreasing prevalence of combustible cigarette use and the increasing prevalence of e-cigarette usage, a trend more pronounced in males than females (Foxon et al., 2024). A similar pattern has also been reported in England (Tattan-Birch et al., 2024). Our study further indicated that dual users, compared to exclusive users, are younger and possess higher education levels and income quintiles. This may suggest a greater propensity for health-seeking behaviors within this group (Jiao et al., 2023). However, dual users also exhibit characteristics such as obesity, high stress levels, and increased alcohol consumption. Further investigation is warranted to determine whether these traits are shared by the demographic groups previously mentioned or are specific to dual users.

Combustible cigarette is recognized as a significant risk factor for cardiovascular diseases, including stroke and angina pectoris, consistent with established literature (Magnussen et al., 2023). Our findings indicate an elevated risk for cardiovascular disease in dual users; however, this result lacks statistical significance, likely due to the small sample size and younger demographic of this group (Osei et al., 2019). While previous studies suggest that dual users may have a higher risk for cardiovascular disease, our study found no significant difference in risk compared to exclusive combustible cigarette users (Choi et al., 2021). Additionally, previous study indicated hypertension, type 2 diabetes, and dyslipidemia are significantly linked to cardiovascular risk show a notably higher association in dual users compared to exclusive combustible cigarette users (Kim et al., 2020). Exclusive NNTP use has also been linked to an elevated risk of metabolic syndrome relative to non-tobacco users (Cai and Bidulescu, 2023). Therefore, the long-term effects of concurrent combustible cigarette and NNTP use on cardiovascular disease require further investigation.

The association between NNTP use and an increased risk of allergic rhinitis, especially among adolescents, is well-documented (Chung et al., 2020). Additionally, adults exposed to NNTP also face an elevated risk (Rha et al., 2022). Notably, the age of smoking initiation is more strongly associated with atopic dermatitis, which is a chronic allergic disorder related to allergic rhinitis (Kantor et al., 2016), than type of tobacco use or amount. This suggests that the younger age profile of dual users may contribute to their increased risk of allergic rhinitis (Oh et al., 2025).

In the United States, adults with depression consistently use cigarettes at higher rates than those without depression (Han et al., 2022). While no significant difference in depression risk was observed between NNTP users and non-tobacco users (Wiernik et al., 2019), stratified analyses by combustible cigarette use revealed a significant association between NNTP use and depression. Prior studies did not compare the depression risk of dual users of combustible cigarette and NNTP to exclusive combustible cigarette users, but our findings indicate that dual users exhibit a significantly higher risk of depression than exclusive combustible cigarette users (Obisesan et al., 2019).

Combustible cigarette is recognized for releasing toxic substances, including nicotine, tar, and carbon monoxide. Concurrently, generating reactive oxygen species stimulates the immune system, releasing pro-inflammatory cytokines and establishing a chronic inflammatory state (Rose et al., 2023). This mechanism explains why smoking is a risk factor for various inflammatory diseases (Agache et al., 2024). Additionally, combustible cigarette use increases sympathetic tone, resulting in vasoconstriction and related symptoms that elevate the risk of cardiovascular disease (Morris et al., 2015).

Current evidence suggests that NNTP similarly induces acute vasoconstriction, impaired exercise tolerance, and other cardiovascular symptoms shortly after exposure (Jin et al., 2021). These physiological responses indicate that NNTP may also contribute to cardiovascular risk, although further research is needed to determine its precise impact relative to combustible cigarette. Both smoking modalities share critical mechanisms, such as vascular dysfunction and increased systemic inflammation, which likely underlie the risks associated with cardiovascular and inflammatory diseases.

Nicotine, the primary component of combustible cigarette and NNTP, stimulates the brain in the short term, alleviating stress, anxiety, and depression (Schroeder and Hoffman, 2014). However, prolonged exposure to nicotine can make the cerebral dopamine pathway more vulnerable, potentially increasing sensitivity and susceptibility in individuals (Piirtola et al., 2021). This may contribute to the heightened risk of depression associated with smoking. However, further research is necessary to understand the stronger correlation between depression and dual users compared to exclusive combustible cigarette users.

This study has several limitations. First, as a cross-sectional study, it can only evaluate associations rather than establish causal relationships. However, based on existing knowledge of NNTP exposure and its effects on the body, we outlined a plausible mechanism by which NNTP may pose long-term risks. Second, reliance on survey data introduces potential biases, such as recall bias, and is subject to the limitations inherent in questionnaire-based data collection. Nonetheless, given the substantial participant pool representative of South Korea, we believe our findings are sufficiently reliable. Third, while the prevalence of NNTP use is anticipated to increase, leading to a growing proportion of exclusive NNTP users, this study primarily focused on comparing exclusive combustible cigarette users and dual users, given the observed shift in prevalence from the former to the latter. Analysis of exclusive NNTP users was not undertaken in this study due to their limited numbers in our dataset, which restricted the statistical power and precision of the estimates, making it challenging to draw reliable conclusions. Future research should aim to include exclusive NNTP users for a more comprehensive analysis of types of tobacco use. Finally, the study could not provide a quantitative assessment of NNTP use or differentiate between types of NNTP, such as nicotine vaping products and heated tobacco products, which limits the evaluation of their individual health impacts. Although we did not present results reflecting the types of NNTP, we believe our study is significant in highlighting the potential risks associated with the concurrent use of NNTP and combustible cigarette.

Despite these limitations, the study has several notable strengths. This is the first study in South Korea to comprehensively assess the characteristics and health risks of dual users using both combustible cigarette and NNTP, offering a novel insight into the shifting smoking patterns. The large, nationally representative sample strengthens the generalizability of the findings, ensuring they are applicable to the broader South Korean population. Furthermore, the study's findings provide valuable data for public health policymakers, emphasizing the need for targeted interventions to address the rising trend of dual user. In addition, while our study could not examine the long-term health impacts of NNTP and dual user, it provides a critical foundation for future research, suggesting important avenues for longitudinal studies. These studies could track the transition to exclusive NNTP use over time and evaluate the health outcomes of various NNTP types. This would be crucial for understanding the differential risks associated with nicotine vaping products versus heated tobacco products.

Additionally, our study identified associations between various diseases in both exclusive combustible cigarette users and dual users, emphasizing the need for caution regarding dual uses. As smoking behaviors evolve, it is vital to continue monitoring these trends and their potential health outcomes. Further research exploring the individual health impacts of different types of NNTP, as well as long-term studies on dual uses, will be essential to inform public health policies and smoking cessation programs. Additionally, as the first study to document the increasing prevalence of individuals using both combustible cigarette and NNTP in South Korea, this research underscores the necessity of examining the long-term health consequences of this emerging trend.

## 5 Conclusion

This study utilized large-scale nationwide survey data from South Korea to investigate trends in types of tobacco use, identifying a decrease in exclusive combustible cigarette users and an increase in dual users. Dual users were found to have distinct sociodemographic characteristics and indicated stronger associations with depression, atopic dermatitis, and allergic rhinitis compared to exclusive combustible cigarette users. These findings challenge the perception that NNTPs are a safer alternative and highlight the potential health risks of concurrent use. Further research is warranted to clarify the long-term effects of NNTPs and their role in smoking cessation. As dual use continues to increase, particularly among younger individuals, targeted public health interventions and regulatory measures are essential to reduce health risks and enhance cessation outcomes.

## CRediT authorship contribution statement

**Tae Hyeon Kim:** Writing – original draft, Investigation. **Yeona Jo:** Writing – original draft, Visualization, Investigation, Formal analysis, Conceptualization. **Jaewon Kim:** Writing – review & editing. **Krishna Prasad Acharya:** Writing – review & editing. **Hanseul Cho:** Writing – review & editing. **Ho Geol Woo:** Writing – review & editing. **Jiyoung Hwang:** Writing – review & editing. **Dong Keon Yon:** Supervision.

## Ethical statement

The KNHANES database was anonymized, and all study participants provided informed consent for participation. The study protocol was approved by the Institutional Review Board of the KDCA (approval numbers: 2007-02CON-04-P, 2008-04EXP-01-C, 2009-01CON-03-2C, 2010-02CON-21-C, 2011-02CON-06-C, 2012-01EXP-01-2C, 2013-07CON-03-4C, 2013-12EXP-03-5C, 2018-01-03-P-A, 2018-01-03-C-A, 2018-01-03-2C-A, and 2018-01-03-5C-A) and by ethical review from the Institutional Review Board of Kyung Hee University. In addition, this study was conducted in compliance with the principles of the Declaration of Helsinki.

## Funding

This research was supported by the Basic Science Research Program through the National Research Foundation of Korea (NRF), funded by the Ministry of Education (RS-2024-00460379). Additionally, this work was supported by the Institute of Information & Communications Technology Planning & Evaluation (IITP) grant funded by the Korea government (MSIT) (RS-2024-00509257, Global AI Frontier Lab). The funders played no role in the study design, data collection, data analysis, data interpretation, or manuscript writing.

## Declaration of competing interest

The authors declare that they have no known competing financial interests or personal relationships that could have appeared to influence the work reported in this paper.

## Data Availability

Data will be made available on request.

## Appendix A Supplementary data

Supplementary material Image 1
