# Supplementary material for: Prevalence and health outcomes of combustible cigarettes and noncombustible nicotine or tobacco products: a nationwide cross-sectional study in South Korea
Source: Prev Med Rep. 2025 Aug 10;57:103202. doi: 10.1016/j.pmedr.2025.103202 (PMC12362025; doi:10.1016/j.pmedr.2025.103202)
Supplement: Supplementary material [file mmc1.docx]

| **Supplementary Material** |
| --- |

Original Article

**Prevalence and health outcomes of combustible cigarettes and noncombustible nicotine or tobacco products: a nationwide cross-sectional study in South Korea**

**Running title:** Type of tobacco use and health outcome

Tae Hyeon Kim,^1,2,3†^ Yeona Jo,^1,3†^ Jaewon Kim,^1^ Krishna Prasad Acharya,^4^ Hanseul Cho,^5^ Ho Geol Woo,^1,2,6^ Jiyoung Hwang,^1,2*^ Dong Keon Yon^1,2,3,4,8*^

^†^ These authors contributed equally to this work as first authors.

* These authors contributed equally to this work as corresponding authors.

***Corresponding authors**

**Jiyoung Hwang**, PhD

Center for Digital Health, Medical Science Research Institute, Kyung Hee University College of Medicine, 23 Kyungheedae–ro, Dongdaemun–gu, Seoul 02447, South Korea

Email: cindy.jyhwang@gmail.com

**Dong Keon Yon**, MD, PhD, FAAAAI, FACAAI, ATSF (lead contact)

Department of Pediatrics, Kyung Hee University Medical Center, Kyung Hee University College of Medicine, 23 Kyungheedae-ro, Dongdaemun-gu, Seoul 02447, Republic of Korea

E-mail: yonkkang@gmail.com

**Contents of Supplementary Material**

| **Figure S1** | Participant selection flow diagram for analysis of tobacco use type and health outcomes among adults in the Korea National Health and Nutrition Examination Survey, 2013–2021. |
| --- | --- |
| **Table S1** | National trends in the prevalence of exclusive combustible cigarette users among Korean adults in the Korean National Health and Nutrition Examination Survey, 2013–2021. |
| **Table S2** | National trends in the prevalence of dual users among Korean adults in the Korean National Health and Nutrition Examination Survey, 2013–2021. |
| **Table S3** | Comparison of health outcome associations between exclusive combustible cigarette users and dual users among Korean adults in the Korea National Health and Nutrition Examination Survey, 2013–2021. |

**Figure S1.** Participant selection flow diagram for analysis of tobacco use type and health outcomes among adults in the Korea National Health and Nutrition Examination Survey, 2013–2021.

*Abbreviations*: BMI, body mass index; CC, combustible cigarette; NNTP, non-combustible nicotine or tobacco products.


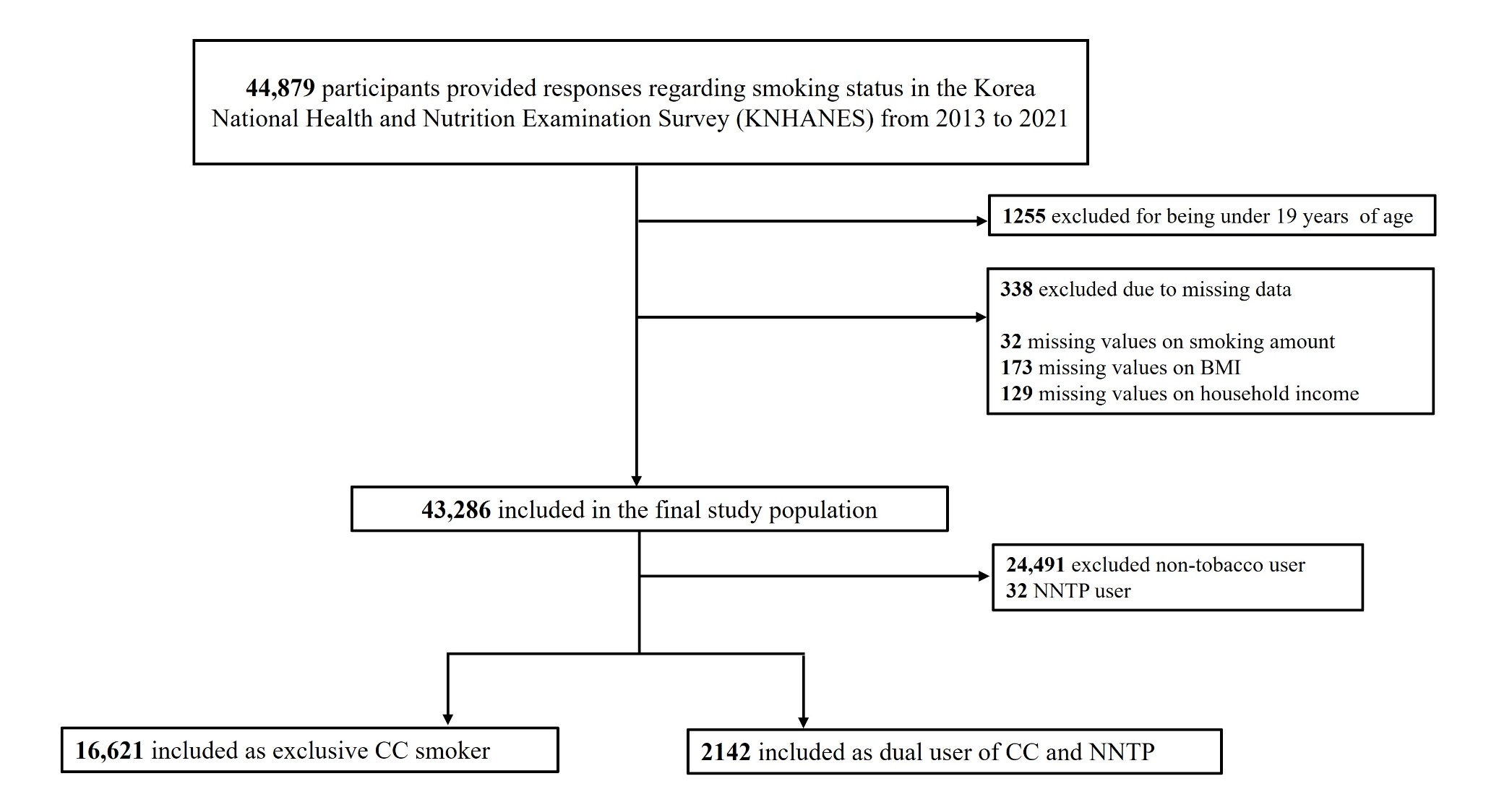


**Table S1.** National trends in the prevalence of exclusive combustible cigarette users among Korean adults in the Korean National Health and Nutrition Examination Survey, 2013–2021.

| **Characteristic** | **Weighted % (95% CI)** | | | | | | | | |
| --- | --- | --- | --- | --- | --- | --- | --- | --- | --- |
|  | 2013 (n=1955) | 2014 (n=1741) | 2015 (n=1743) | 2016 (n=2103) | 2017 (n=2110) | 2018 (n=2044) | 2019 (n=1737) | 2020 (n=1623) | 2021 (n=1565) |
| **Overall** | 46.11 (44.53 to 47.69) | 43.59 (41.74 to 45.43) | 41.75 (40.11 to 43.39) | 44.40 (42.62 to 46.18) | 43.26 (41.59 to 44.93) | 40.15 (38.56 to 41.74) | 33.04 (31.40 to 34.68) | 34.18 (32.60 to 35.76) | 33.90 (32.18 to 35.62) |
| **Age group, years** |  |  |  |  |  |  |  |  |  |
| 19–39 | 43.31 (40.32 to 46.29) | 38.47 (35.00 to 41.95) | 34.65 (31.72 to 37.57) | 40.27 (36.99 to 43.56) | 37.25 (34.18 to 40.32) | 32.63 (29.75 to 35.51) | 21.03 (18.42 to 23.63) | 21.90 (19.29 to 24.51) | 22.35 (19.98 to 24.72) |
| 40–64 | 47.74 (45.52 to 49.97) | 46.85 (44.43 to 49.27) | 46.50 (44.30 to 48.70) | 47.24 (45.16 to 49.32) | 47.14 (45.03 to 49.24) | 44.94 (42.98 to 46.90) | 38.88 (36.74 to 41.02) | 40.54 (38.29 to 42.79) | 37.55 (34.94 to 40.17) |
| ≥65 | 49.65 (46.16 to 53.14) | 48.49 (45.21 to 51.77) | 46.75 (43.22 to 50.28) | 46.09 (42.85 to 49.34) | 46.51 (43.58 to 49.44) | 44.20 (41.03 to 47.37) | 44.63 (41.07 to 48.20) | 45.31 (42.13 to 48.50) | 50.14 (47.04 to 53.24) |
| **Sex** |  |  |  |  |  |  |  |  |  |
| Male | 75.11 (72.99 to 77.23) | 72.53 (69.80 to 75.27) | 70.15 (67.83 to 72.46) | 73.37 (71.19 to 75.55) | 71.45 (69.19 to 73.71) | 64.72 (62.54 to 66.90) | 53.95 (51.20 to 56.69) | 55.02 (52.48 to 57.56) | 53.92 (51.49 to 56.34) |
| Female | 13.74 (12.07 to 15.42) | 12.76 (10.82 to 14.69) | 11.47 (9.98 to 12.95) | 12.71 (10.99 to 14.43) | 12.35 (10.89 to 13.81) | 13.28 (11.46 to 15.10) | 10.38 (8.96 to 11.79) | 10.65 (9.18 to 12.12) | 11.43 (9.49 to 13.38) |
| **Region of residence** |  |  |  |  |  |  |  |  |  |
| Urban | 45.47 (43.75 to 47.19) | 42.46 (40.44 to 44.47) | 41.27 (39.46 to 43.08) | 43.84 (41.96 to 45.71) | 42.73 (40.94 to 44.51) | 39.33 (37.61 to 41.05) | 32.05 (30.22 to 33.88) | 33.48 (31.76 to 35.20) | 32.58 (30.67 to 34.49) |
| Rural | 49.14 (45.14 to 53.13) | 49.96 (45.48 to 54.44) | 44.32 (39.99 to 48.65) | 47.76 (42.50 to 53.02) | 46.49 (42.42 to 50.57) | 45.74 (42.11 to 49.37) | 38.34 (34.70 to 41.98) | 38.44 (33.71 to 43.17) | 41.55 (38.14 to 44.97) |
| **Educational level** |  |  |  |  |  |  |  |  |  |
| Elementary school or lower education | 37.83 (34.14 to 41.51) | 37.52 (33.60 to 41.44) | 39.56 (35.77 to 43.36) | 34.23 (30.05 to 38.40) | 34.23 (30.79 to 37.67) | 31.44 (27.49 to 35.39) | 33.21 (29.08 to 37.34) | 32.09 (27.30 to 36.89) | 36.73 (32.84 to 40.63) |
| Middle school | 48.00 (42.67 to 53.34) | 45.14 (40.02 to 50.27) | 42.83 (38.25 to 47.41) | 48.19 (43.38 to 53.00) | 50.59 (45.24 to 55.94) | 46.08 (40.79 to 51.37) | 43.71 (38.32 to 49.10) | 43.50 (38.64 to 48.35) | 44.02 (38.46 to 49.58) |
| High school | 49.95 (47.21 to 52.68) | 49.91 (46.43 to 53.40) | 46.00 (42.59 to 49.41) | 47.81 (44.55 to 51.07) | 48.57 (45.52 to 51.62) | 47.39 (44.40 to 50.38) | 38.72 (35.52 to 41.93) | 41.84 (39.07 to 44.62) | 40.72 (37.42 to 44.02) |
| College or higher education | 45.39 (42.54 to 48.24) | 41.08 (38.37 to 43.80) | 39.65 (37.18 to 42.12) | 44.07 (41.68 to 46.46) | 41.23 (38.94 to 43.52) | 36.97 (35.00 to 38.95) | 28.77 (26.70 to 30.85) | 29.43 (27.37 to 31.48) | 28.73 (26.67 to 30.79) |
| **Household income** |  |  |  |  |  |  |  |  |  |
| Lowest quartile | 49.52 (45.58 to 53.46) | 47.46 (42.69 to 52.23) | 50.23 (46.42 to 54.04) | 42.78 (38.72 to 46.85) | 45.02 (41.08 to 48.96) | 41.54 (37.60 to 45.49) | 37.44 (33.68 to 41.20) | 42.50 (37.76 to 47.23) | 39.97 (35.78 to 44.16) |
| Second quartile | 47.45 (44.51 to 50.40) | 43.76 (40.01 to 47.51) | 42.51 (38.67 to 46.35) | 45.66 (42.49 to 48.84) | 44.30 (41.34 to 47.26) | 41.30 (37.90 to 44.71) | 35.08 (32.13 to 38.04) | 37.83 (34.67 to 40.98) | 36.74 (33.35 to 40.13) |
| Third quartile | 46.16 (43.09 to 49.23) | 44.36 (41.39 to 47.33) | 40.02 (37.11 to 42.93) | 44.81 (42.03 to 47.60) | 44.16 (41.38 to 46.94) | 41.83 (38.94 to 44.71) | 34.05 (31.33 to 36.77) | 31.34 (28.44 to 34.25) | 34.61 (31.52 to 37.69) |
| Highest quartile | 43.42 (40.44 to 46.41) | 41.13 (37.93 to 44.32) | 39.42 (36.50 to 42.35) | 43.84 (40.83 to 46.85) | 41.00 (38.32 to 43.67) | 37.06 (34.45 to 39.66) | 29.15 (26.35 to 31.95) | 31.78 (29.72 to 33.85) | 29.52 (26.78 to 32.26) |
| **BMI group*** |  |  |  |  |  |  |  |  |  |
| Underweight | 34.17 (27.03 to 41.31) | 37.99 (29.70 to 46.28) | 26.89 (19.61 to 34.17) | 37.27 (27.33 to 47.21) | 32.58 (25.21 to 39.96) | 29.16 (20.71 to 37.62) | 18.95 (12.89 to 25.02) | 23.50 (16.73 to 30.28) | 23.31 (16.22 to 30.40) |
| Normal weight | 40.80 (38.41 to 43.19) | 37.01 (34.21 to 39.80) | 34.06 (31.32 to 36.80) | 37.35 (34.47 to 40.23) | 37.09 (34.36 to 39.83) | 34.32 (31.84 to 36.79) | 28.24 (25.75 to 30.73) | 28.25 (25.98 to 30.51) | 28.23 (25.34 to 31.11) |
| Overweight | 48.71 (45.41 to 52.01) | 46.15 (41.99 to 50.32) | 46.92 (43.20 to 50.63) | 47.04 (43.25 to 50.84) | 47.05 (43.91 to 50.19) | 44.22 (40.54 to 47.90) | 37.60 (34.45 to 40.75) | 36.95 (33.46 to 40.43) | 36.90 (33.48 to 40.33) |
| Obese | 53.12 (49.98 to 56.26) | 51.44 (48.59 to 54.30) | 49.29 (46.28 to 52.30) | 51.23 (48.57 to 53.88) | 49.03 (46.43 to 51.64) | 45.32 (42.62 to 48.02) | 37.19 (34.50 to 39.89) | 38.84 (36.14 to 41.53) | 38.85 (35.92 to 41.78) |
| **Marital status** |  |  |  |  |  |  |  |  |  |
| Married | 46.57 (44.96 to 48.17) | 45.53 (43.77 to 47.29) | 43.72 (41.97 to 45.46) | 45.52 (43.89 to 47.15) | 44.91 (43.28 to 46.54) | 41.86 (40.18 to 43.53) | 36.50 (34.60 to 38.40) | 38.16 (36.48 to 39.84) | 37.54 (35.43 to 39.65) |
| Unmarried | 44.62 (40.36 to 48.88) | 37.42 (32.66 to 42.18) | 35.93 (32.14 to 39.71) | 40.86 (36.17 to 45.54) | 38.33 (34.27 to 42.39) | 35.11 (31.60 to 38.62) | 22.28 (19.40 to 25.17) | 23.88 (20.40 to 27.37) | 24.45 (21.59 to 27.31) |
| **Subjective health level** |  |  |  |  |  |  |  |  |  |
| High | 44.50 (41.23 to 47.77) | 42.80 (39.83 to 45.76) | 43.03 (39.84 to 46.23) | 42.68 (39.28 to 46.08) | 41.91 (38.73 to 45.10) | 37.57 (34.83 to 40.32) | 31.64 (29.17 to 34.10) | 33.23 (30.48 to 35.99) | 32.52 (30.27 to 34.78) |
| Middle | 46.97 (44.63 to 49.30) | 43.41 (40.76 to 46.05) | 39.92 (37.40 to 42.44) | 44.67 (42.43 to 46.91) | 44.07 (41.83 to 46.32) | 41.11 (38.99 to 43.24) | 34.11 (31.76 to 36.45) | 34.54 (32.29 to 36.79) | 33.71 (30.99 to 36.43) |
| Low | 46.98 (42.81 to 51.15) | 45.83 (41.81 to 49.85) | 44.72 (40.89 to 48.55) | 46.69 (42.97 to 50.42) | 42.89 (38.80 to 46.98) | 42.00 (38.10 to 45.91) | 32.47 (28.63 to 36.31) | 34.80 (30.96 to 38.65) | 37.49 (34.09 to 40.89) |
| **Subjective stress level** |  |  |  |  |  |  |  |  |  |
| High | 49.50 (46.05 to 52.95) | 45.08 (41.70 to 48.47) | 42.33 (38.94 to 45.72) | 47.52 (44.44 to 50.61) | 42.79 (39.68 to 45.90) | 41.98 (38.83 to 45.13) | 30.96 (28.08 to 33.83) | 33.21 (30.24 to 36.19) | 32.62 (29.10 to 36.14) |
| Middle | 44.98 (42.98 to 46.97) | 42.86 (40.50 to 45.22) | 40.90 (38.86 to 42.94) | 43.25 (41.07 to 45.43) | 43.61 (41.47 to 45.76) | 38.43 (36.19 to 40.66) | 33.45 (31.21 to 35.69) | 33.92 (31.92 to 35.91) | 33.69 (31.49 to 35.90) |
| Low | 45.51 (41.31 to 49.71) | 43.83 (39.71 to 47.95) | 43.99 (39.85 to 48.13) | 42.87 (38.71 to 47.03) | 42.76 (38.95 to 46.58) | 43.20 (39.11 to 47.28) | 35.81 (31.55 to 40.07) | 37.77 (32.94 to 42.60) | 37.03 (33.19 to 40.87) |
| **Alcohol consumption, days/month** |  |  |  |  |  |  |  |  |  |
| <1 | 30.43 (28.11 to 32.75) | 26.91 (24.38 to 29.43) | 27.56 (24.96 to 30.15) | 28.08 (25.50 to 30.66) | 27.99 (25.68 to 30.30) | 28.47 (26.16 to 30.77) | 23.49 (21.26 to 25.71) | 25.25 (22.86 to 27.64) | 27.11 (24.70 to 29.52) |
| 1–4 | 43.69 (40.87 to 46.50) | 42.68 (39.77 to 45.60) | 39.45 (36.81 to 42.09) | 41.63 (38.88 to 44.39) | 39.72 (37.08 to 42.35) | 37.00 (34.12 to 39.88) | 31.54 (28.96 to 34.12) | 31.11 (28.46 to 33.75) | 31.41 (28.60 to 34.23) |
| ≥5 | 71.48 (68.74 to 74.23) | 67.21 (64.33 to 70.10) | 65.82 (62.27 to 69.37) | 70.09 (67.29 to 72.90) | 68.08 (65.42 to 70.75) | 61.20 (58.12 to 64.29) | 50.33 (46.88 to 53.79) | 53.07 (49.51 to 56.64) | 50.75 (47.01 to 54.49) |
| **Diagnosis of stroke** |  |  |  |  |  |  |  |  |  |
| No | 45.89 (44.29 to 47.49) | 43.41 (41.52 to 45.29) | 41.42 (39.80 to 43.04) | 44.20 (42.38 to 46.03) | 42.93 (41.23 to 44.64) | 39.88 (38.27 to 41.48) | 32.66 (31.02 to 34.30) | 33.94 (32.36 to 35.53) | 33.56 (31.84 to 35.27) |
| Yes | 58.03 (47.63 to 68.42) | 56.07 (45.60 to 66.54) | 61.18 (49.11 to 73.26) | 56.75 (46.50 to 67.00) | 62.29 (51.73 to 72.85) | 57.86 (47.51 to 68.21) | 55.60 (44.15 to 67.06) | 50.40 (39.97 to 60.83) | 55.16 (43.59 to 66.73) |
| **Diagnosis of angina pectoris** |  |  |  |  |  |  |  |  |  |
| No | 46.06 (44.49 to 47.64) | 43.55 (41.68 to 45.42) | 41.63 (39.98 to 43.29) | 44.12 (42.30 to 45.93) | 42.97 (41.29 to 44.66) | 39.83 (38.23 to 41.44) | 32.67 (31.02 to 34.33) | 33.82 (32.22 to 35.43) | 33.61 (31.86 to 35.36) |
| Yes | 50.21 (36.20 to 64.22) | 46.66 (32.31 to 61.01) | 51.16 (39.05 to 63.28) | 62.72 (52.48 to 72.97) | 65.40 (54.03 to 76.76) | 64.72 (54.20 to 75.24) | 57.48 (46.87 to 68.09) | 57.23 (44.14 to 70.32) | 56.76 (45.25 to 68.26) |
| **Diagnosis of hypertension** |  |  |  |  |  |  |  |  |  |
| No | 45.49 (43.68 to 47.30) | 42.78 (40.68 to 44.88) | 39.83 (37.94 to 41.73) | 42.85 (40.83 to 44.86) | 41.28 (39.35 to 43.21) | 38.22 (36.42 to 40.01) | 30.38 (28.59 to 32.17) | 30.92 (29.19 to 32.65) | 30.78 (28.81 to 32.76) |
| Yes | 49.71 (46.39 to 53.02) | 48.31 (44.71 to 51.91) | 51.31 (47.55 to 55.07) | 51.23 (47.79 to 54.67) | 52.62 (49.38 to 55.86) | 49.10 (45.90 to 52.30) | 44.65 (41.25 to 48.05) | 48.58 (45.47 to 51.69) | 46.86 (43.64 to 50.09) |
| **Diagnosis of dyslipidemia** |  |  |  |  |  |  |  |  |  |
| No | 46.32 (44.65 to 47.98) | 43.58 (41.64 to 45.51) | 42.10 (40.30 to 43.91) | 43.88 (41.86 to 45.91) | 43.24 (41.35 to 45.13) | 39.63 (37.79 to 41.47) | 31.90 (30.05 to 33.75) | 32.34 (30.59 to 34.09) | 31.86 (29.93 to 33.79) |
| Yes | 44.31 (39.95 to 48.68) | 43.67 (38.58 to 48.77) | 39.08 (34.94 to 43.23) | 47.70 (43.77 to 51.62) | 43.35 (39.53 to 47.16) | 43.21 (39.90 to 46.51) | 39.17 (35.40 to 42.93) | 43.22 (39.80 to 46.64) | 43.00 (39.80 to 46.21) |
| **Diagnosis of type 2 diabetes mellitus** |  |  |  |  |  |  |  |  |  |
| No | 45.52 (43.80 to 47.25) | 42.63 (40.70 to 44.56) | 41.14 (39.43 to 42.86) | 43.50 (41.67 to 45.33) | 42.52 (40.77 to 44.27) | 39.38 (37.67 to 41.08) | 32.14 (30.43 to 33.86) | 33.02 (31.37 to 34.67) | 31.90 (30.06 to 33.74) |
| Yes | 54.67 (48.52 to 60.81) | 58.81 (53.73 to 63.89) | 51.88 (46.17 to 57.59) | 56.15 (50.89 to 61.40) | 53.88 (48.16 to 59.60) | 48.97 (43.51 to 54.42) | 44.50 (39.18 to 49.82) | 47.85 (42.23 to 53.47) | 55.11 (50.01 to 60.22) |
| **Diagnosis of rheumatoid arthritis** |  |  |  |  |  |  |  |  |  |
| No | 46.28 (44.65 to 47.91) | 43.60 (41.73 to 45.46) | 41.83 (40.19 to 43.48) | 44.56 (42.77 to 46.34) | 43.41 (41.70 to 45.11) | 40.30 (38.69 to 41.91) | 33.10 (31.45 to 34.75) | 34.31 (32.72 to 35.90) | 33.93 (32.19 to 35.67) |
| Yes | 35.00 (22.15 to 47.85) | 42.30 (28.49 to 56.12) | 36.17 (23.13 to 49.21) | 34.06 (21.92 to 46.21) | 33.24 (22.63 to 43.86) | 27.78 (16.17 to 39.39) | 29.07 (18.73 to 39.41) | 23.12 (11.61 to 34.63) | 31.27 (19.18 to 43.37) |
| **Diagnosis of allergic rhinitis** |  |  |  |  |  |  |  |  |  |
| No | 47.36 (45.64 to 49.09) | 45.11 (43.17 to 47.05) | 42.26 (40.43 to 44.09) | 45.85 (43.97 to 47.72) | 44.96 (43.09 to 46.83) | 41.06 (39.35 to 42.77) | 34.86 (33.13 to 36.59) | 35.46 (33.74 to 37.18) | 35.37 (33.39 to 37.34) |
| Yes | 38.90 (34.53 to 43.27) | 34.29 (29.03 to 39.56) | 39.30 (35.36 to 43.24) | 36.81 (32.90 to 40.73) | 34.64 (30.53 to 38.75) | 35.27 (31.28 to 39.26) | 24.09 (20.65 to 27.53) | 28.15 (24.82 to 31.48) | 26.96 (22.74 to 31.19) |
| **Diagnosis of atopic dermatitis** |  |  |  |  |  |  |  |  |  |
| No | 46.21 (44.56 to 47.85) | 44.13 (42.26 to 45.99) | 41.75 (40.11 to 43.39) | 44.65 (42.86 to 46.44) | 43.27 (41.54 to 44.99) | 40.82 (39.22 to 42.42) | 33.53 (31.86 to 35.19) | 34.55 (32.90 to 36.20) | 34.56 (32.77 to 36.35) |
| Yes | 43.36 (32.73 to 53.99) | 27.16 (18.70 to 35.61) | 58.31 (49.01 to 67.60) | 37.55 (27.60 to 47.50) | 43.00 (33.51 to 52.50) | 25.28 (17.20 to 33.36) | 20.45 (14.63 to 26.27) | 26.62 (19.51 to 33.73) | 20.39 (13.84 to 26.95) |
| **Diagnosis of depression** |  |  |  |  |  |  |  |  |  |
| No | 46.36 (44.74 to 47.99) | 43.84 (41.98 to 45.69) | 42.09 (40.39 to 43.78) | 44.73 (42.93 to 46.53) | 43.40 (41.68 to 45.11) | 40.17 (38.56 to 41.77) | 33.22 (31.52 to 34.91) | 34.19 (32.55 to 35.83) | 34.13 (32.39 to 35.88) |
| Yes | 38.76 (30.91 to 46.62) | 38.84 (30.22 to 47.47) | 33.76 (25.80 to 41.72) | 37.31 (29.66 to 44.95) | 38.92 (30.54 to 47.30) | 39.75 (32.03 to 47.48) | 28.81 (22.11 to 35.51) | 34.04 (27.74 to 40.34) | 29.26 (21.75 to 36.77) |

Abbreviations: BMI, body mass index; CI, confidence interval.

* According to Asian-Pacific guidelines, BMI is divided into four groups: underweight (<18.5 kg/m^2^), normal weight (18.5–22.9 kg/m^2^),

overweight (23.0–24.9 kg/m^2^), and obese (≥25.0 kg/m^2^).**Table S2.** National trends in the prevalence of dual users among Korean adults in the Korean National Health and Nutrition Examination Survey, 2013–2021.

| **Characteristic** | **Weighted % (95% CI)** | | | | | | | | |
| --- | --- | --- | --- | --- | --- | --- | --- | --- | --- |
|  | 2013 (n=39) | 2014 (n=69) | 2015 (n=121) | 2016 (n=90) | 2017 (n=89) | 2018 (n=256) | 2019 (n=559) | 2020 (n=463) | 2021 (n=456) |
| **Overall** | 1.05 (0.64 to 1.46) | 2.29 (1.63 to 2.94) | 3.89 (3.04 to 4.74) | 2.19 (1.68 to 2.70) | 2.31 (1.74 to 2.88) | 6.20 (5.25 to 7.15) | 13.08 (11.73 to 14.42) | 12.33 (11.02 to 13.63) | 12.76 (11.39 to 14.13) |
| **Age group, years** |  |  |  |  |  |  |  |  |  |
| 19–39 | 1.12 (0.45 to 1.79) | 3.92 (2.58 to 5.26) | 6.29 (4.53 to 8.06) | 3.43 (2.43 to 4.42) | 4.36 (3.03 to 5.69) | 9.68 (7.85 to 11.52) | 21.58 (18.76 to 24.40) | 22.02 (19.34 to 24.70) | 20.66 (18.13 to 23.19) |
| 40–64 | 1.13 (0.55 to 1.71) | 1.38 (0.80 to 1.97) | 2.70 (1.87 to 3.52) | 1.68 (1.08 to 2.29) | 1.22 (0.76 to 1.67) | 5.06 (3.99 to 6.13) | 9.81 (8.38 to 11.25) | 7.94 (6.56 to 9.32) | 10.35 (8.81 to 11.89) |
| ≥65 | 0.49 (0.03 to 0.95) | 0.17 (0.00 to 0.38) | 0.62 (0.11 to 1.13) | 0.34 (0.00 to 0.74) | 0.37 (0.05 to 0.68) | 0.55 (0.00 to 1.11) | 1.79 (0.93 to 2.64) | 1.15 (0.42 to 1.88) | 1.35 (0.58 to 2.13) |
| **Sex** |  |  |  |  |  |  |  |  |  |
| Male | 1.75 (1.04 to 2.46) | 4.12 (2.90 to 5.33) | 6.47 (5.02 to 7.92) | 3.83 (2.89 to 4.77) | 3.76 (2.83 to 4.68) | 10.51 (8.90 to 12.12) | 21.36 (19.17 to 23.54) | 19.49 (17.48 to 21.50) | 20.58 (18.38 to 22.78) |
| Female | 0.27 (0.02 to 0.52) | 0.34 (0.07 to 0.61) | 1.14 (0.58 to 1.70) | 0.40 (0.14 to 0.66) | 0.73 (0.26 to 1.19) | 1.48 (1.01 to 1.94) | 4.11 (3.01 to 5.20) | 4.24 (3.16 to 5.31) | 3.97 (2.99 to 4.96) |
| **Region of residence** |  |  |  |  |  |  |  |  |  |
| Urban | 1.05 (0.60 to 1.50) | 2.41 (1.68 to 3.14) | 3.93 (3.05 to 4.81) | 2.13 (1.59 to 2.67) | 2.39 (1.75 to 3.02) | 6.53 (5.46 to 7.59) | 13.94 (12.50 to 15.38) | 12.96 (11.52 to 14.39) | 13.57 (12.04 to 15.10) |
| Rural | 1.04 (0.03 to 2.05) | 1.59 (0.20 to 2.98) | 3.67 (1.27 to 6.07) | 2.54 (1.18 to 3.91) | 1.84 (0.62 to 3.06) | 3.94 (2.12 to 5.76) | 8.41 (5.54 to 11.28) | 8.49 (5.75 to 11.24) | 8.05 (5.64 to 10.46) |
| **Educational level** |  |  |  |  |  |  |  |  |  |
| Elementary school or lower education | 0.13 (0.00 to 0.38) | 0.21 (0.00 to 0.55) | 1.30 (0.09 to 2.50) | 0.92 (0.16 to 1.67) | 0.23 (0.00 to 0.57) | NA | 1.34 (0.24 to 2.44) | 0.27 (0.00 to 0.80) | 1.17 (0.00 to 2.68) |
| Middle school | 1.90 (0.18 to 3.62) | 0.66 (0.00 to 1.46) | 3.74 (1.03 to 6.46) | 1.03 (0.01 to 2.05) | 0.38 (0.00 to 1.00) | 2.75 (0.56 to 4.94) | 5.34 (2.79 to 7.89) | 2.16 (0.52 to 3.79) | 2.20 (0.40 to 3.99) |
| High school | 1.60 (0.67 to 2.53) | 2.24 (1.17 to 3.30) | 4.34 (2.71 to 5.97) | 2.87 (1.85 to 3.90) | 1.86 (0.94 to 2.78) | 5.78 (4.20 to 7.37) | 12.01 (9.74 to 14.28) | 10.54 (8.33 to 12.75) | 12.04 (9.68 to 14.40) |
| College or higher education | 0.77 (0.25 to 1.28) | 3.06 (2.03 to 4.09) | 4.23 (3.06 to 5.40) | 2.28 (1.55 to 3.01) | 3.20 (2.29 to 4.10) | 7.99 (6.51 to 9.48) | 16.39 (14.40 to 18.38) | 15.98 (14.20 to 17.76) | 16.02 (14.12 to 17.93) |
| **Household income** |  |  |  |  |  |  |  |  |  |
| Lowest quartile | 0.72 (0.00 to 1.50) | 0.54 (0.00 to 1.24) | 3.76 (1.65 to 5.86) | 1.38 (0.46 to 2.29) | 2.02 (0.57 to 3.47) | 3.31 (1.66 to 4.96) | 9.26 (6.18 to 12.35) | 6.92 (4.33 to 9.51) | 5.84 (3.80 to 7.89) |
| Second quartile | 0.93 (0.11 to 1.74) | 1.84 (0.79 to 2.88) | 3.59 (1.98 to 5.20) | 2.26 (1.30 to 3.22) | 1.51 (0.44 to 2.58) | 5.68 (4.00 to 7.36) | 11.52 (9.46 to 13.58) | 11.07 (8.56 to 13.59) | 12.14 (9.27 to 15.02) |
| Third quartile | 1.41 (0.49 to 2.32) | 3.45 (2.00 to 4.90) | 5.02 (3.17 to 6.86) | 2.18 (1.26 to 3.11) | 2.58 (1.49 to 3.68) | 6.50 (4.78 to 8.22) | 15.39 (12.86 to 17.92) | 12.70 (10.52 to 14.88) | 13.72 (11.14 to 16.31) |
| Highest quartile | 0.98 (0.42 to 1.54) | 2.12 (1.10 to 3.14) | 3.09 (1.65 to 4.53) | 2.51 (1.50 to 3.52) | 2.73 (1.64 to 3.82) | 7.58 (6.03 to 9.12) | 13.74 (11.52 to 15.95) | 14.50 (12.32 to 16.69) | 14.51 (12.22 to 16.79) |
| **BMI group *** |  |  |  |  |  |  |  |  |  |
| Underweight | 0.18 (0.00 to 0.53) | 2.94 (0.00 to 6.42) | 6.37 (1.04 to 11.70) | NA | 2.82 (0.00 to 5.72) | 0.84 (0.00 to 2.02) | 7.94 (3.23 to 12.66) | 7.76 (4.22 to 11.31) | 9.30 (4.91 to 13.69) |
| Normal weight | 0.84 (0.30 to 1.38) | 2.00 (1.19 to 2.82) | 3.17 (2.11 to 4.22) | 1.62 (0.85 to 2.39) | 1.43 (0.79 to 2.07) | 4.46 (3.48 to 5.44) | 10.93 (8.94 to 12.92) | 9.03 (7.14 to 10.91) | 9.70 (7.92 to 11.49) |
| Overweight | 1.26 (0.39 to 2.14) | 1.66 (0.56 to 2.75) | 3.15 (1.55 to 4.75) | 2.31 (1.34 to 3.28) | 2.05 (1.02 to 3.08) | 6.56 (4.70 to 8.42) | 13.21 (10.72 to 15.70) | 13.82 (11.39 to 16.26) | 12.76 (9.83 to 15.69) |
| Obese | 1.31 (0.52 to 2.09) | 3.02 (1.67 to 4.38) | 4.91 (3.29 to 6.53) | 2.99 (2.01 to 3.98) | 3.42 (2.33 to 4.50) | 8.49 (6.43 to 10.55) | 16.06 (13.82 to 18.30) | 14.80 (12.74 to 16.86) | 16.13 (13.70 to 18.56) |
| **Marital status** |  |  |  |  |  |  |  |  |  |
| Married | 0.96 (0.53 to 1.39) | 1.45 (0.94 to 1.97) | 2.73 (2.02 to 3.44) | 1.53 (1.07 to 1.98) | 1.65 (1.17 to 2.12) | 4.89 (3.95 to 5.83) | 9.71 (8.44 to 10.97) | 8.65 (7.46 to 9.84) | 10.03 (8.65 to 11.40) |
| Unmarried | 1.35 (0.41 to 2.30) | 4.92 (2.83 to 7.01) | 7.33 (4.81 to 9.84) | 4.29 (2.71 to 5.87) | 4.29 (2.59 to 5.99) | 10.05 (7.78 to 12.31) | 23.54 (19.94 to 27.15) | 21.84 (18.89 to 24.79) | 19.85 (16.84 to 22.86) |
| **Subjective health level** |  |  |  |  |  |  |  |  |  |
| High | 1.00 (0.35 to 1.64) | 1.07 (0.34 to 1.80) | 3.35 (2.11 to 4.59) | 2.03 (1.04 to 3.03) | 2.00 (0.97 to 3.03) | 3.53 (2.42 to 4.64) | 14.13 (11.97 to 16.29) | 10.90 (8.85 to 12.95) | 12.07 (10.23 to 13.91) |
| Middle | 1.07 (0.53 to 1.61) | 2.94 (1.94 to 3.95) | 4.01 (2.98 to 5.03) | 2.12 (1.45 to 2.80) | 2.25 (1.50 to 3.00) | 7.22 (5.96 to 8.48) | 12.26 (10.62 to 13.91) | 13.02 (11.22 to 14.81) | 14.19 (12.30 to 16.09) |
| Low | 1.11 (0.10 to 2.11) | 2.64 (1.13 to 4.14) | 4.54 (2.38 to 6.70) | 2.66 (1.50 to 3.83) | 3.04 (1.34 to 4.74) | 8.04 (5.60 to 10.48) | 13.53 (10.13 to 16.93) | 12.82 (9.70 to 15.94) | 10.04 (7.28 to 12.81) |
| **Subjective stress level** |  |  |  |  |  |  |  |  |  |
| High | 0.80 (0.27 to 1.33) | 2.82 (1.38 to 4.26) | 6.56 (4.76 to 8.37) | 3.00 (2.03 to 3.96) | 3.37 (2.08 to 4.66) | 7.43 (5.59 to 9.26) | 17.76 (15.28 to 20.24) | 17.64 (14.86 to 20.42) | 17.02 (14.27 to 19.78) |
| Middle | 1.22 (0.59 to 1.85) | 2.34 (1.45 to 3.22) | 2.93 (1.99 to 3.87) | 2.07 (1.38 to 2.77) | 1.91 (1.26 to 2.57) | 6.28 (5.08 to 7.48) | 11.47 (9.87 to 13.07) | 10.50 (9.21 to 11.79) | 11.80 (10.22 to 13.37) |
| Low | 0.74 (0.11 to 1.37) | 1.15 (0.00 to 2.34) | 1.83 (0.52 to 3.14) | 1.03 (0.19 to 1.86) | 1.66 (0.54 to 2.77) | 3.72 (2.14 to 5.29) | 9.84 (7.13 to 12.54) | 8.14 (5.17 to 11.10) | 8.92 (5.86 to 11.99) |
| **Alcohol consumption, days/month** |  |  |  |  |  |  |  |  |  |
| <1 | 0.28 (0.01 to 0.54) | 1.54 (0.70 to 2.37) | 1.27 (0.38 to 2.16) | 1.17 (0.49 to 1.85) | 1.16 (0.45 to 1.86) | 2.85 (1.96 to 3.75) | 5.55 (4.29 to 6.82) | 6.04 (4.76 to 7.32) | 7.03 (5.50 to 8.55) |
| 1–4 | 0.83 (0.30 to 1.36) | 2.30 (1.28 to 3.33) | 4.13 (2.82 to 5.43) | 2.82 (1.88 to 3.76) | 2.56 (1.60 to 3.53) | 6.51 (5.06 to 7.95) | 14.36 (12.21 to 16.51) | 14.51 (12.08 to 16.93) | 12.76 (10.59 to 14.92) |
| ≥5 | 2.45 (1.11 to 3.80) | 3.27 (1.95 to 4.59) | 7.27 (5.39 to 9.16) | 2.59 (1.72 to 3.47) | 3.44 (2.22 to 4.65) | 10.52 (8.31 to 12.73) | 22.60 (19.56 to 25.64) | 19.11 (16.34 to 21.88) | 23.43 (20.11 to 26.76) |
| **Diagnosis of stroke** |  |  |  |  |  |  |  |  |  |
| No | 1.07 (0.65 to 1.49) | 2.29 (1.63 to 2.96) | 3.92 (3.06 to 4.77) | 2.23 (1.71 to 2.74) | 2.35 (1.77 to 2.93) | 6.25 (5.29 to 7.22) | 13.15 (11.79 to 14.50) | 12.37 (11.07 to 13.67) | 12.88 (11.50 to 14.25) |
| Yes | NA | 1.70 (0.00 to 4.19) | 2.29 (0.00 to 5.65) | NA | NA | 2.65 (0.00 to 7.80) | 8.92 (1.78 to 16.06) | 9.14 (0.00 to 19.10) | 5.40 (0.54 to 10.26) |
| **Diagnosis of angina pectoris** |  |  |  |  |  |  |  |  |  |
| No | 1.03 (0.61 to 1.45) | 2.31 (1.65 to 2.97) | 3.93 (3.06 to 4.79) | 2.22 (1.71 to 2.74) | 2.34 (1.76 to 2.92) | 6.28 (5.31 to 7.24) | 13.17 (11.81 to 14.52) | 12.39 (11.08 to 13.70) | 12.89 (11.51 to 14.27) |
| Yes | 2.36 (0.00 to 5.77) | NA | 1.02 (0.00 to 3.00) | NA | NA | NA | 7.06 (0.82 to 13.30) | 8.37 (0.00 to 20.82) | 2.24 (0.00 to 5.02) |
| **Diagnosis of hypertension** |  |  |  |  |  |  |  |  |  |
| No | 1.19 (0.70 to 1.67) | 2.52 (1.76 to 3.28) | 4.33 (3.34 to 5.32) | 2.44 (1.85 to 3.04) | 2.47 (1.84 to 3.11) | 6.72 (5.65 to 7.79) | 14.34 (12.84 to 15.84) | 13.84 (12.34 to 15.33) | 14.03 (12.44 to 15.61) |
| Yes | 0.27 (0.00 to 0.69) | 0.92 (0.09 to 1.75) | 1.69 (0.72 to 2.66) | 1.08 (0.41 to 1.74) | 1.53 (0.65 to 2.41) | 3.76 (2.29 to 5.23) | 7.56 (5.57 to 9.56) | 5.67 (3.82 to 7.52) | 7.48 (5.31 to 9.65) |
| **Diagnosis of dyslipidemia** |  |  |  |  |  |  |  |  |  |
| No | 1.02 (0.59 to 1.45) | 2.33 (1.62 to 3.05) | 4.28 (3.33 to 5.23) | 2.40 (1.83 to 2.97) | 2.45 (1.82 to 3.08) | 6.54 (5.47 to 7.61) | 13.99 (12.50 to 15.48) | 13.50 (12.02 to 14.98) | 14.13 (12.67 to 15.58) |
| Yes | 1.30 (0.00 to 2.63) | 1.87 (0.28 to 3.46) | 0.96 (0.24 to 1.68) | 0.84 (0.17 to 1.52) | 1.51 (0.24 to 2.77) | 4.18 (2.52 to 5.85) | 8.17 (5.89 to 10.44) | 6.56 (4.35 to 8.77) | 6.66 (4.38 to 8.95) |
| **Diagnosis of type 2 diabetes mellitus** |  |  |  |  |  |  |  |  |  |
| No | 1.04 (0.61 to 1.47) | 2.33 (1.65 to 3.02) | 3.97 (3.08 to 4.85) | 2.26 (1.73 to 2.80) | 2.39 (1.79 to 2.98) | 6.33 (5.33 to 7.33) | 13.12 (11.70 to 14.54) | 12.89 (11.52 to 14.27) | 13.36 (11.91 to 14.81) |
| Yes | 1.18 (0.00 to 2.82) | 1.53 (0.00 to 3.23) | 2.58 (0.19 to 4.98) | 1.23 (0.12 to 2.34) | 1.23 (0.12 to 2.34) | 4.27 (1.41 to 7.14) | 12.52 (8.50 to 16.54) | 5.69 (2.95 to 8.42) | 6.34 (3.44 to 9.23) |
| **Diagnosis of rheumatoid arthritis** |  |  |  |  |  |  |  |  |  |
| No | 1.07 (0.65 to 1.49) | 2.31 (1.65 to 2.98) | 3.87 (3.01 to 4.73) | 2.22 (1.71 to 2.74) | 2.34 (1.77 to 2.92) | 6.19 (5.22 to 7.16) | 13.23 (11.86 to 14.59) | 12.39 (11.07 to 13.71) | 12.84 (11.47 to 14.21) |
| Yes | NA | NA | 5.40 (0.00 to 12.85) | NA | NA | 6.73 (0.00 to 14.82) | 3.39 (0.00 to 7.18) | 6.96 (0.65 to 13.28) | 5.62 (0.49 to 10.75) |
| **Diagnosis of allergic rhinitis** |  |  |  |  |  |  |  |  |  |
| No | 1.09 (0.62 to 1.56) | 2.24 (1.53 to 2.95) | 3.82 (2.92 to 4.72) | 2.10 (1.56 to 2.64) | 2.18 (1.58 to 2.77) | 5.86 (4.85 to 6.88) | 12.53 (11.11 to 13.95) | 12.06 (10.66 to 13.46) | 11.70 (10.27 to 13.13) |
| Yes | 0.84 (0.09 to 1.58) | 2.55 (0.93 to 4.18) | 4.22 (2.13 to 6.30) | 2.68 (1.36 to 4.00) | 2.99 (1.46 to 4.52) | 7.99 (5.74 to 10.23) | 15.74 (12.78 to 18.71) | 13.60 (10.64 to 16.56) | 17.77 (14.26 to 21.29) |
| **Diagnosis of atopic dermatitis** |  |  |  |  |  |  |  |  |  |
| No | 1.01 (0.59 to 1.44) | 2.27 (1.61 to 2.93) | 3.84 (2.99 to 4.69) | 2.17 (1.65 to 2.70) | 2.24 (1.66 to 2.83) | 5.91 (4.99 to 6.84) | 12.95 (11.60 to 14.31) | 12.05 (10.78 to 13.31) | 12.54 (11.17 to 13.91) |
| Yes | 2.14 (0.00 to 4.54) | 2.86 (0.00 to 6.33) | 5.30 (0.87 to 9.74) | 2.63 (0.00 to 5.59) | 4.09 (0.40 to 7.78) | 12.49 (6.87 to 18.12) | 16.19 (9.74 to 22.65) | 18.08 (11.82 to 24.34) | 17.30 (10.60 to 24.00) |
| **Diagnosis of depression** |  |  |  |  |  |  |  |  |  |
| No | 1.05 (0.62 to 1.47) | 2.20 (1.57 to 2.84) | 3.90 (3.03 to 4.77) | 2.24 (1.71 to 2.76) | 2.25 (1.68 to 2.81) | 6.17 (5.22 to 7.12) | 13.19 (11.81 to 14.57) | 12.22 (10.94 to 13.50) | 12.73 (11.36 to 14.11) |
| Yes | 1.12 (0.00 to 2.69) | 3.85 (0.41 to 7.30) | 3.64 (0.00 to 7.36) | 1.16 (0.00 to 2.80) | 4.30 (0.00 to 8.91) | 6.92 (2.93 to 10.90) | 10.39 (5.45 to 15.33) | 14.49 (9.33 to 19.65) | 13.26 (8.56 to 17.96) |

Abbreviations: BMI, body mass index; CI, confidence interval; NNTP, noncombustible nicotine or tobacco product.

* According to Asian-Pacific guidelines, BMI is divided into four groups: underweight (<18.5 kg/m^2^), normal weight (18.5–22.9 kg/m^2^),

overweight (23.0–24.9 kg/m^2^), and obese (≥25.0 kg/m^2^).

**Table S3.** Comparison of health outcome associations between exclusive combustible cigarette users and dual users among Korean adults in the Korea National Health and Nutrition Examination Survey, 2013–2021.

| **Health outcomes** | **Smoking type** | **aOR (95% CI)** | **crude OR (95% CI)** |
| --- | --- | --- | --- |
| **Stroke** | Exclusive combustible cigarette users | 1.00 | 1.00 |
|  | Dual users | 0.90 (0.54 to 1.51) | 2.84 (2.35 to 3.43) |
| **Angina pectoris** | Exclusive combustible cigarette users | 1.00 | 1.00 |
|  | Dual users | 0.74 (0.33 to 1.64) | 3.00 (2.47 to 3.65) |
| **Hypertension** | Exclusive combustible cigarette users | 1.00 | 1.00 |
|  | Dual users | 0.86 (0.73 to 1.02) | 2.39 (2.08 to 2.74) |
| **Dyslipidemia** | Exclusive combustible cigarette users | 1.00 | 1.00 |
|  | Dual users | 0.99 (0.84 to 1.18) | 1.84 (1.59 to 2.13) |
| **Type 2 diabetes** | Exclusive combustible cigarette users | 1.00 | 1.00 |
|  | Dual users | 1.03 (0.83 to 1.30) | 2.01 (1.68 to 2.40) |
| **Rheumatoid arthritis** | Exclusive combustible cigarette users | 1.00 | 1.00 |
|  | Dual users | 1.02 (0.58 to 1.78) | 2.13 (1.75 to 2.58) |
| **Allergic rhinitis** | Exclusive combustible cigarette users | 1.00 | 1.00 |
|  | Dual users | 1.30 (1.14 to 1.49) | 0.96 (0.86 to 1.06) |
| **Atopic dermatitis** | Exclusive combustible cigarette users | 1.00 | 1.00 |
|  | Dual users | 1.39 (1.10 to 1.74) | 0.97 (0.84 to 1.12) |
| **Depression** | Exclusive combustible cigarette users | 1.00 | 1.00 |
|  | Dual users | 1.50 (1.19 to 1.89) | 1.48 (1.27 to 1.72) |

Abbreviations: CC, combustible cigarette; CI, confidence interval; aOR, adjusted odds ratio; OR, odds ratio.

The numbers in bold indicate significant differences (P < 0.05).
